# Current approaches addressing oral health practitioners’ responsiveness to child abuse and neglect: A scoping review protocol

**DOI:** 10.1371/journal.pone.0296650

**Published:** 2024-02-08

**Authors:** Heuiwon Han, Jane Koziol-McLain, Zac Morse, Amanda B. Lees

**Affiliations:** 1 Department of Oral Health, School of Clinical Sciences, Auckland University of Technology, Auckland, New Zealand; 2 Centre for Interdisciplinary Trauma Research, Auckland University of Technology, Auckland, New Zealand; 3 School of Public Health and Interdisciplinary Studies, Auckland University of Technology, Auckland, New Zealand; Xiamen University - Malaysia Campus: Xiamen University - Malaysia, MALAYSIA

## Abstract

**Introduction:**

Child abuse and neglect (CAN) poses significant risks, causing severe and long-lasting effects on a child’s well-being, including physical and mental health and learning and socializing capabilities. Oral health practitioners (OHPs) uniquely position themselves to identify signs of maltreatment in the orofacial area, offer appropriate support, and collaborate with a multidisciplinary team. The literature has shown that OHPs under-report child protection concerns to a statutory child protection agency. Responding to CAN is often hindered by various factors, such as the fear of making false accusations and insufficient knowledge to detect and report potential cases. However, the literature lacks a comprehensive understanding of the strategies and interventions that can address the responsiveness of OHPs and other professionals to child protection issues. This scoping review aims to provide a broad overview and map the literature on the existing approaches to enhance the responsiveness of OHPs in child protection.

**Materials and methods:**

The proposed scoping review will be conducted following the JBI methodology for scoping reviews guideline and reported using the PRISMA-ScR guideline. The first exploratory search is conducted to refine the search strategy and inclusion and exclusion criteria. The second search will include MEDLINE (EBSCO), CINAHL (EBSCO), Dentistry & Oral Science Source (EBSCO), Cochrane Library, and Scopus, with a date range from January 2000 to March 2023. The third search will involve reference list searching and gray literature searching in Google and Google Scholar. Government and international health organizations’ websites will be searched for policies and guidelines. The review will consider studies that report the current approaches to address OHPs’ responsiveness to CAN in any setting. Two reviewers will independently select sources and extract data. Any disagreements will be resolved by consensus of the research team. The extracted data will be presented in a tabulated chart with a narrative summary.

## Introduction

Child Abuse and neglect (CAN) can severely impact children’s social development, academic achievements, and both their short-term and long-term physical and mental well-being [1–5].

Injuries resulting from CAN range from fractures and lacerations to central nervous system damage and may also heighten the risk of emotional developmental impairments, sexual dysfunction, and depression [2,3,5–9]. Oral health practitioners (OHPs) play a crucial role in protecting children from CAN [6,10–12]. They are skilled in identifying orofacial manifestations of CAN [4,13,14] and can offer support to affected children and their families and refer suspected cases to child protection agencies when necessary [15].

Despite the critical role that OHPs can play in protecting children from abuse and neglect, there is a significant global issue of under-reporting suspected cases. A survey of 510 Croatian dentists revealed that while 26.27% had suspected CAN cases during their careers, only 42.9% of those reported their concerns [16]. Furthermore, only 11.4% of these respondents were aware of the proper reporting procedure, and approximately 70% indicated a need for additional training [16]. A similar pattern was observed in New Zealand, where a study of 92 dental and oral health therapists found that 62% had encountered at least one suspected case during their careers. However, only 21% reported their concerns [12]. Although 74% of the respondents believed they could easily recognize signs and symptoms of CAN, only 48% were familiar with the reporting process [12].

Recent reviews have investigated how OHPs respond to CAN concerns and the barriers they face in these situations [17,18]. However, these reviews did not focus on the approaches to enhance OHP’s responsiveness. One study highlighted the uncertainty and hesitation OHPs often experience when identifying and reporting suspected cases [18]. Common barriers identified include fear of parental reprisal towards the child, potential violence towards the practitioner, uncertainty in diagnosis, and lack of knowledge about reporting processes [18,19]. These findings underscore concerns regarding current interdisciplinary practices, such as strained relationships with social services and inconsistent policies and guidelines [18].

To enhance OHPs’ responsiveness to CAN, developing and implementing early intervention and prevention strategies is essential. Integrating such strategies into clinical practices would promote a proactive and collaborative approach, enhancing the ability to identify and address potential cases of CAN. While specific strategies, such as multidisciplinary team approaches, exist [20], they need to be tailored to the unique context of OHPs, who often operate in isolation. This isolation can hinder collaborative efforts with other healthcare professionals, emphasizing the need for a deeper understanding of existing strategies.

Considering the lack of detailed studies on strategies for improving OHPs’ responsiveness towards child protection, a scoping review is an appropriate method to comprehensively map evidence across the topic [21,22]. This type of review is particularly suited for this purpose as it has not been extensively explored in the current literature. The objective of this scoping review is to systematically map the literature on the approaches utilized by OHPs to enhance the responsiveness of OHPs in child protection. This will include exploring various approaches, such as education programs, clinical protocols, and professional collaboration pathways aimed at strengthening the role of OHPs in safeguarding child welfare.

## Materials and methods

The planned scoping review will adhere to the Joanna Briggs Institute (JBI) methodology for scoping reviews guideline [23] and reported using the Preferred Reporting Items for Systematic Reviews and Meta-Analyses extension for Scoping Reviews (PRISMA-ScR) guideline (S1 Table) [24]. The JBI methodology for scoping review guideline [23] is highly regarded for its clear scope definition, rigorous and transparent processes, and ability to incorporate diverse evidence types. Structured data extraction and synthesis processes, outlined in the JBI guideline [23], facilitate the derivation of impactful conclusions relevant to practice and policy.

The PCC (Population, Concept, Context) framework [23] was used to develop search strategies and inclusion and exclusion criteria. This framework ensures a precise and transparent definition of eligibility criteria, facilitating a clear understanding of the study’s scope and promoting replicability in future research [23,25]. Any changes made to the protocol will be reported along with the scoping review findings.

### Inclusion and exclusion criteria

This scoping review is guided by the question: What are the current approaches to address the responsiveness of oral health practitioners in child protection?. The JBI PCC (Population, Concept, Context) framework [23] was used to establish inclusion and exclusion criteria (S2 Table).

The ’population’ was defined as registered OHPs or students in undergraduate and postgraduate programs training to become OHPs. Registered OHPs and undergraduate and postgraduate students will be considered as the review will focus on the broad application of child protection strategies in oral health, applicable universally across the profession. The review is planned for the entire oral health community, with the understanding that the strategies for enhancing responsiveness do not vary between the professional statuses of the individuals. The population includes general dentists, pediatric dentists, pedodontists, other dental specialists, oral health therapists, dental therapists, dental hygienists, dental nurses, orthodontic auxiliaries, or any equivalent profession.

The ’concept’ is the current approaches used to improve the responsiveness of OHPs in child protection. This encompasses any efforts and strategies for preventing and responding to child abuse and neglect, including the identification, reporting, and appropriate handling of cases. Current approaches include but are not limited to policy statements on implementing the interdisciplinary practice, an in-service professional education program to train OHPs on identifying and reporting suspected CAN cases, or guidelines to help OHPs to identify and report suspected CAN cases. Studies will not be included if they solely describe the current state of detection or reporting without discussing strategies or interventions. Additionally, studies that offer future recommendations without basing them on existing methods will not be included. An individual up to the age of 18 will be considered a child for this review.

The ’context’ will include all settings, such as private and public dental clinics, school-based dental clinics, or university training clinics.

Articles published only in English will be considered due to expertise and financial resource limitations. The search date range will be from January 2000 to April 2023 to ensure the findings are contemporary to current OHPs. Literature, including OHPs as a part of broader health practitioners, will be excluded from this scoping review unless data is segregated by discipline.

### Search strategy

This scoping review will include primary studies with quantitative, qualitative, and mixed-methods designs, as well as systematic reviews and meta-analyses that will be used to identify primary sources. Discussion papers, editorials, and government and international health organizations’ policy documents will also be considered. Discussion papers and editorials offer valuable insights into current practices, trends, and debates in oral health and child protection, enhancing the review’s contextual depth. Policy documents from government and international health organizations are crucial for understanding the regulatory frameworks and best practices guiding practitioners in child protection. To identify relevant literature, a three-step search method recommended by the JBI scoping review guideline [22] will be implemented across five databases.

An initial exploratory search was conducted in MEDLINE (via EBSCO) and CINAHL (via EBSCO) to identify key articles for understanding the potential scope of the review. Key terms contained in the titles, abstracts, keywords, and subject headings of relevant literature were used to develop a comprehensive search strategy (see search strategy for MEDLINE, CINAHL, Dentistry & Oral Sciences Source via EBSCO in Table 1). A second search will be conducted across five databases (MEDLINE via EBSCO, CINAHL via EBSCO, & Dentistry & Oral Science Source via EBSCO, Cochrane Library, and Scopus). All identified keywords will be adapted for each included database. A third search will involve exploring the reference list of identified sources. Authors of primary sources or reviews will be contacted if further information is required. Additionally, the first 100 items from Google Scholar and Google will be explored with the search terms for any gray literature not indexed in the stated databases, including relevant government and international health organizations’ policy documents.

**Table 1:** Search strategy for MEDLINE, CINAHL, & dentistry & oral sciences source via EBSCO.

Number	Query (Limit Year = January 2000 to March 2023)
1.	“dental professional*” or “dental practitioner*” or “oral health practitioner*” or dentist* or “dental specialist*” or pedodontist* or “oral health therapist*” or “dental therapist*” or “dental hygienist*” or “orthodontic auxilliar*”
2.	child* or adolescen* or youth or teen* or “young people” or “young person*” or kid* or paediatric* or pediatric*
3.	abus* or neglect* or maltreat* or safeguard*
4.	report* or respond* or respons* or detect* or act* or react* or approach* or alert* or deal*
5.	strateg* or intervention* or policies or policy or framework* or guideline* or training* or education or program* or approach*
6	1 AND 2 AND 3 AND 4 AND 5

Keyword searching – All Fields using EBSCO Health Databases.

### Source of evidence selection

Following the search, all identified citations will be collated and uploaded into the reference management software EndNote v.X9 (Clarivate Analytics, PA, USA), and duplicates will be removed. Subsequently, the records will be uploaded into the web-based review software tool Covidence (Veritas Health Innovation, Melbourne, Australia). Titles and abstracts will be independently screened by two reviewers for assessment against the inclusion and exclusion criteria of the review. The full texts of selected citations will then be assessed in detail against the inclusion and exclusion criteria by two independent reviewers. Both the title and abstract screening and the full-text screening will be piloted before the complete review of all literature. Reasons for excluding sources of evidence at the full-text screening stage will be recorded and reported in the scoping review. Any disagreements that arise between two reviewers at each stage of the selection process will be resolved through discussion with the research team. The search results and the study inclusion process will be reported in full in the final scoping review and presented in a PRISMA-ScR flow diagram [24].

### Data extraction

Data will be extracted from selected reports independently by two reviewers using the data extraction table, and then the research team will combine two data sets. Data extracted will include details about the population, concept, context, study methods and key findings relevant to current approaches to improve the responsiveness of OHPs to child protection concerns. The data extraction table (S3 Table) is adapted from the JBI methodology for scoping review guideline [23] and modified to suit the specific requirements of this study, ensuring a more targeted and relevant analysis of the selected sources. To ensure consistency and accuracy, the research team will pilot the form with three sample sources. The research team will discuss any differences encountered, and the form will be modified as necessary. Any modification made will be detailed when reporting the scoping review findings. In applying the modified extraction form, any disagreements between the reviewers will be resolved through discussion with the research team.

### Data analysis and presentation

Qualitative content analysis [26] will be implemented to summarize the informational content of the data, which will be presented in both narrative summaries and tables. The current approaches will initially be categorized into various groups based on their concepts, such as pre-service and in-service professional education, implementation of interdisciplinary practice policies, and coordination of multidisciplinary approaches. The identified approaches will not be segregated by professional status (professions or students) but will be presented as uniformly applicable practices for enhancing child protection responsiveness in oral health.

These categories will be updated as analysis of the data progreeses. The initial set of categories will then be revised, merging smaller categories and splitting larger ones to align with the review question. A final table, based on refined categories, will map out the diverse efforts and strategies employed to improve the responsiveness of OHPs to child protection concerns. The data table will be complemented by narrative summaries [23]. A final report will be reported following the PRISMA-ScR guideline, and the potential implications of the findings for further research, practice, and policy development will be discussed [27].

## Supporting information

S1 Table Inclusion and exclusion criteria. (DOCX)

S2 Table Data extraction form. (DOCX)

S3 Table Preferred reporting items for systematic reviews and meta-analyses extension for scoping reviews checklist. (PDF)
