# Supplementary material for: Current approaches addressing oral health practitioners’ responsiveness to child abuse and neglect: A scoping review protocol
Source: PLoS One. 2024 Feb 8;19(2):e0296650. doi: 10.1371/journal.pone.0296650 (PMC10852231; doi:10.1371/journal.pone.0296650)
Supplement: S1 Table — (DOCX) [file pone.0296650.s001.docx]

**S1 Table. Inclusion and exclusion criteria.**

| Criteria | Inclusion | Exclusion |
| --- | --- | --- |
| Population | - Oral health practitioners who are registered with national regulatory bodies (general dentists, pediatric dental specialists, pedodontists, other dental specialists, oral health therapists, dental therapists, dental hygienists, and orthodontic auxiliaries) - Undergraduate and postgraduate students for dental-related programs | - Non-registered oral health providers - Oral health practitioners as a part of broader health practitioners (unless data is segregated by discipline) |
| Concept | - Child abuse and neglect response strategies - Child protection response strategies - Current approaches include but are not limited to implementing interdisciplinary practice policies or introducing pre-service or in-service professional education | - Only reporting the current detecting and reporting status - Only reporting barriers to responding to child abuse and neglect concerns - Future recommendations only rather than current practice |
| Context | - All international dental-related settings, including private and public dental services - Dental services provided in community settings such as schools |  |
| Type of sources | - Primary studies, including quantitative, qualitative, and mix-methods study designs - Systematic reviews and meta-analysis - Discussion papers, editorials, and government and international health organizations’ policy documents - English, full-text - Publication year: January 2000 to March 2023 | - Book reviews, book chapters, news articles, commentaries, letters, legal judgments |
