# Supplementary material for: Current approaches addressing oral health practitioners’ responsiveness to child abuse and neglect: A scoping review protocol
Source: PLoS One. 2024 Feb 8;19(2):e0296650. doi: 10.1371/journal.pone.0296650 (PMC10852231; doi:10.1371/journal.pone.0296650)
Supplement: S2 Table — (DOCX) [file pone.0296650.s002.docx]

**S2 Table. Data extraction form.**

| **Source information – Citation No.** | |
| --- | --- |
| Title |  |
| Primary author |  |
| Year of publication |  |
| Citation (APA 7th) |  |
| Primary author location |  |
| Industry funding (Y/N/NS) – If Y, specify the role of funders in the study |  |
| Type of literature (article, policy document, editorial, etc) |  |
| **General information (complete any part that is applicable)** | |
| Aim and objectives |  |
| Study design |  |
| Methods |  |
| **Population (complete any part that is applicable)** | |
| Study population or target population |  |
| Sample size and response rate |  |
| **Context (complete any part that is applicable)** | |
| Setting (country, public dental sector, private dental sector, hospital, etc) |  |
| **Concept (complete any part that is applicable)** | |
| Current approaches to address the responsiveness |  |
| Findings or results |  |
| Identified barriers |  |
| Identified facilitators |  |
| Identified needs or OHPs |  |
| Any interdisciplinary collaboration (Y/N) – If Y, describe collaboration including disciplines and how |  |
| Any address to equity and culture (Y/N) – If Y, describe efforts or strategies to address equity and culture |  |
| **Other Findings** | |
| Any further recommendation |  |
| Reported strength or limitations |  |
| Other comments |  |

Abbreviations: Y – yes, N – No, NS – Not specified
